# Predictors of opioid misuse in patients with chronic pain: a prospective cohort study

**DOI:** 10.1186/1472-6963-6-46

**Published:** 2006-04-04

**Authors:** Timothy J Ives, Paul R Chelminski, Catherine A Hammett-Stabler, Robert M Malone, J Stephen Perhac, Nicholas M Potisek, Betsy Bryant Shilliday, Darren A DeWalt, Michael P Pignone

**Affiliations:** 1 Division of General Internal Medicine and Clinical Epidemiology, Department of Medicine, School of Medicine, University of North Carolina at Chapel Hill, Chapel Hill, North Carolina, USA; 2 Division of Pharmacotherapy and Experimental Therapeutics, School of Pharmacy, University of North Carolina at Chapel Hill, Chapel Hill, North Carolina, USA; 3 Department of Pathology and Laboratory Medicine, School of Medicine, University of North Carolina at Chapel Hill, Chapel Hill, North Carolina, USA; 4 Center for Excellence in Chronic Illness Care, University of North Carolina Health System, Chapel Hill, North Carolina, USA

## Abstract

**Background:**

Opioid misuse can complicate chronic pain management, and the non-medical use of opioids is a growing public health problem. The incidence and risk factors for opioid misuse in patients with chronic pain, however, have not been well characterized. We conducted a prospective cohort study to determine the one-year incidence and predictors of opioid misuse among patients enrolled in a chronic pain disease management program within an academic internal medicine practice.

**Methods:**

One-hundred and ninety-six opioid-treated patients with chronic, non-cancer pain of at least three months duration were monitored for opioid misuse at pre-defined intervals. Opioid misuse was defined as: 1. Negative urine toxicological screen (UTS) for prescribed opioids; 2. UTS positive for opioids or controlled substances not prescribed by our practice; 3. Evidence of procurement of opioids from multiple providers; 4. Diversion of opioids; 5. Prescription forgery; or 6. Stimulants (cocaine or amphetamines) on UTS.

**Results:**

The mean patient age was 52 years, 55% were male, and 75% were white. Sixty-two of 196 (32%) patients committed opioid misuse. Detection of cocaine or amphetamines on UTS was the most common form of misuse (40.3% of misusers). In bivariate analysis, misusers were more likely than non-misusers to be younger (48 years vs 54 years, p < 0.001), male (59.6% vs. 38%; p = 0.023), have past alcohol abuse (44% vs 23%; p = 0.004), past cocaine abuse (68% vs 21%; p < 0.001), or have a previous drug or DUI conviction (40% vs 11%; p < 0.001%). In multivariate analyses, age, past cocaine abuse (OR, 4.3), drug or DUI conviction (OR, 2.6), and a past alcohol abuse (OR, 2.6) persisted as predictors of misuse. Race, income, education, depression score, disability score, pain score, and literacy were not associated with misuse. No relationship between pain scores and misuse emerged.

**Conclusion:**

Opioid misuse occurred frequently in chronic pain patients in a pain management program within an academic primary care practice. Patients with a history of alcohol or cocaine abuse and alcohol or drug related convictions should be carefully evaluated and followed for signs of misuse if opioids are prescribed. Structured monitoring for opioid misuse can potentially ensure the appropriate use of opioids in chronic pain management and mitigate adverse public health effects of diversion.

## Background

The past decade and a half has witnessed an expansion of opioid analgesic use for patients who have chronic non-cancer pain [1-5]. The misuse of opioid analgesics, however, is a growing public health problem [6,7]. National surveys show that opioid misuse has increased dramatically over the past decade and that opioid medications have surpassed cocaine and heroin use as the leading drugs of abuse [8,9]. Utah and North Carolina have documented dramatic increases in unintentional overdose deaths from opioid analgesics diverted from their intended medical use [10,11]. The increased misuse is also reflected in the trauma literature which reports increases in opioid use among patients admitted to trauma centers [12]. As an ongoing response to the long-standing public health problem of prescription drug diversion, (as of May 2005), at least 28 states have established or are in the process of enacting legislation to establish prescription monitoring systems for controlled substances, and the medical literature is beginning to examine their effectiveness [13,14].

Chronic pain is recognized as another important public health problem that is often undertreated [3,15,16]. Experts advocate the use of opioids in a carefully selected "subset" of patients with chronic non-cancer pain, but few data are available to guide selection of patients for whom opioids are likely to have net benefit [1,17]. The limited clinical trial data on opioid use in chronic pain derives mainly from small trials in highly selected patients seen in specialty settings [18-22]. The decision of whether and how providers should use these agents in a primary care setting, however, falls largely on expert opinion and clinical judgment. Generalists are faced with the dilemma of balancing the pain-relieving properties of opioids in selected patients with chronic pain against the reality that some patients may misuse and divert these medications. In effect, they are balancing one public health priority – the relief of suffering from pain – against another, the mitigation of substance misuse.

The incidence and prevalence of opioid misuse in patients treated for chronic pain is unclear and remains a topic of debate. Little is known about the factors predisposing patients to opioid misuse in the outpatient setting. Although histories of drug or alcohol abuse are commonly accepted proxies for patients at risk for opioid abuse [23], few epidemiologic data are available that clearly define risk factors for opioid misuse by chronic pain patients [24]. Most studies have been small (less than 50 patients) or were conducted with patients who were receiving substance abuse treatment, such as patients enrolled in methadone treatment clinics [23-26]. A case-control study of 533 hospitalized patients identified previous substance abuse, ongoing alcohol abuse, and urine toxicological screens positive for opiates as risk factors for misuse, but this study focused on inpatients hospitalized in a drug addiction unit and did not address the question of substance misuse in pain patients [27]. Other studies of misuse conducted in pain specialty clinics have relied on surveys and retrospective chart reviews, but did not monitor patients prospectively for predefined clinical outcomes [28-30]. Generalization of their findings to a primary care setting is limited.

We sought to determine the one-year incidence and predictors of opioid misuse in a cohort of patients enrolled in a chronic pain disease management program within an academic general internal medicine practice.

## Methods

### Patient recruitment

This study was conducted in patients with chronic pain who were referred to a chronic pain disease management program within an academic internal medicine practice [31]. Patients were eligible if they had non-cancer pain of greater than three months duration, and we encouraged referral of patients whose pain was considered difficult to manage and in whom opioid misuse was suspected. Patients were managed by a multidisciplinary team in consultation with the patient's primary care physician. The team was composed of a clinical pharmacist practitioner, an internist, a psychiatrist with sub-specialization in pain medicine, a nurse, and a program assistant. Patients were seen initially at monthly intervals during the medication titration phase. In addition to standard non-pharmacotherapeutic modalities, the use of anti-inflammatory agents, adjunctive analgesics, or long-acting (e.g., methadone) or sustained-release opioid agents (e.g., morphine ER) were preferred. Once patients achieved adequate, stable pain control with a proportionate improvement in function, they were scheduled to return every three months for monitoring of pain, depression, functional status, and misuse.

### Defining and identifying opioid misuse

At enrollment, patients signed a medication agreement at enrollment [32], specifying the conditions under which opioids or controlled substances (O/CS) would be prescribed. Patients agreed to the following:

• To receive O/CS only from this practice.

• To use a single pharmacy.

• Not to sell or share medication.

• Not to abuse alcohol or illicit drugs (e.g. cocaine).

• That lost, stolen, or misplaced medication would generally not be replaced and that consideration of replacement would only occur at a clinic visit.

• That requests for medication renewals would occur only during regular clinic business hours, and not by telephone request.

• That regular urine toxicological screens would be performed, and

• That background checks for criminal drug and alcohol convictions would be performed.

As stipulated in the medication agreement, we prospectively monitored for misuse through clinical history, review of medications, review of outside medical records, communication with pharmacies and providers, and urine toxicological screening (UTS) [33,34]. Prescriptions for O/CS were documented both in the institutional electronic medical record and our disease management program database. Discrepancies and inconsistencies in opioid medication use were discussed with the patient's primary provider. Pharmacies were contacted to verify procurement of O/CS medications, and, if misuse was suspected, additional pharmacies were contacted to ascertain whether or not a patient was receiving opioids from multiple sources.

We defined opioid misuse prospectively as any of the following:

1. Negative UTS: Defined as UTS negative on at least two occasions for prescribed O/CS in the context of a reported history that the patient was taking the medication as prescribed (Repeatedly "negative" urines were considered an indicator of possible diversion)

2. Inconsistent UTS: Defined as UTS positive on at least two occasions for O/CS medications not prescribed by our practice

3. Doctor collecting: Evidence of concurrent procurement of O/CS from multiple providers

4. Diversion of O/CS

5. Prescription forgery

6. Stimulant positive (cocaine or amphetamine) UTS: Evidence of cocaine or amphetamines in the urine while being prescribed opioids was considered opioid misuse because it was in violation of the patient's medication agreement and because concurrent use of cocaine and amphetamines was felt to increase the risk of diversion in order to procure additional stimulants.

Urine toxicology screening included immunoassays for opiates, amphetamines, cannabinoids, benzodiazepines, methadone, propoxyphene, cocaine metabolite, and barbiturates. Testing was conducted at each visit and was correlated with the patient's reported history of O/CS use. In collaboration with our institution's toxicologist, results of the UTS were verified using gas chromatography/mass spectrophotometry (GC/MS) confirmatory assays. Because the UTS *opiate *assay has greater sensitivity and specificity for morphine and codeine, the presence or absence (i.e., inappropriately negative when it should have been positive) of other opiates (i.e., hydromorphone, oxycodone, hydrocodone, oxymorphone) were also confirmed by GC/MS. All positive results for amphetamines were confirmed with GC/MS to exclude the possibility of assay interference from other medications [34]. Urine samples were tested for low urine creatinine levels (i.e., < 20 ng/mL) to detect inappropriately diluted samples.

A single positive cannabinoid finding on UTS was not defined as misuse, but patients with multiple chronic positive results were strongly counseled to refrain from use of marijuana. Continued positive UTS for cannabinoids were tracked, however, to examine this variable as a potential predictor of opioid misuse as defined above. Neither past drug or alcohol abuse, nor past drug or alcohol criminal convictions disqualified patients from participating in our program, or receiving opioids within it.

Patients were advised at entry into the program that that the aforementioned violations would result in discontinuation of O/CS. A formal committee was constituted to evaluate and respond to instances of opioid misuse. It consisted of the practice medical director, two other attending physicians, the program clinical pharmacist practitioner, two resident physicians, and a nurse. The committee deliberated through secure e-mail. Patients committing opioid misuse were offered referral to substance abuse experts at our institution, or in their respective communities. Practice policy stipulated that the reinstitution of O/CS therapy could occur if the patient completed 6 months of substance abuse counseling. Patients who forged prescriptions were subject to dismissal from the practice.

### Predictors of opioid misuse

Patients provided informed consent and underwent a comprehensive baseline medical assessment that included collection of socio-demographic data, assessment of pain, disability, mood, and literacy, using validated scales. Using the 11-point Brief Pain Inventory (BPI), patients rated their current pain and their pain at its worst, least, and average over the past month [35,36]. The seven-item Pain Disability Index (PDI), a measure of pain-related disability, asked patients to rate the degree of disability on a 10-point scale [37-39]. To assess depression, the Center for Epidemiological Studies-Depression Scale (CES-D) was used [40]. This twenty-item tool rates affective symptoms on a scale of 0 to 3. Literacy was measured using Rapid Estimate of Adult Literacy in Medicine (REALM) instrument [41], a word recognition test that assesses reading ability and uses health care terms. Previous history of cocaine or alcohol abuse was assessed by self report. Past criminal convictions for drug, or driving while impaired violations were researched using the publicly accessible database of the North Carolina Department of Correction Public Access Information System [42].

### Analysis

Opioid misuse, as defined above, was the primary outcome of interest. The misuse categories described are presented individually as counts and proportions and are combined as a composite outcome in logistic regression analysis. Predictors of both opioid and other drug misuse were examined in bivariate and multivariate analyses. Bivariate analyses are reported as proportions and relative risks, with p-values and 95% confidence intervals (CI) for dichotomous variables and t-tests for continuous variables. All exposure variables with a p-value of <0.1 were analyzed in multivariate modeling. Models were reduced using the Maximum Likelihood Ratio Test. Statistical analyses were performed using Stata 7.0 (Stata Corporation, College Station, TX). The research protocol was approved by the Committee on the Protection of the Rights of Human Subjects, School of Medicine, University of North Carolina at Chapel Hill and patients provided informed consent.

## Results

Between December 2002 and December 2003, 199 consecutive patients were referred. Of that number, 196 agreed to participate, and were enrolled (Table 1). The mean age was 52 years, 55% were male, 75% were white, 96% were taking opioids, 28% had a history of alcohol abuse, and 28% had a history of cocaine abuse. Eighty-five percent reported an income of less than $20,000 per year. Depression was common: the average CES-D score was 23.6, 74% of patients scored in the depressed range, and 54% scored in the severe depression range. The average literacy score using the REALM was 51.2, and 54% of patients scored below the 9th grade reading level (REALM < 60). The mean PDI score was 45.2, suggesting substantial functional impairment. Twelve percent of patients had North Carolina drug convictions; eleven percent had driving while impaired convictions (DUI); and 20% had either drug or alcohol convictions. Back pain was the most common cause of chronic pain, and the distribution of primary pain types was consistent with other reports of pain types reported in the general medicine literature, with the exception of the under-representation of headache (Data not shown) [28,43,44]. At 12 months, four patients were lost to follow-up, and three changed their venue of primary care.

### Incidence of opioid misuse

Over the one-year study period, opioid misuse occurred in sixty-two (32%) patients (Table 2). Twenty-five patients were found to have positive and confirmed urine drug screens for stimulants; twenty-four were positive for cocaine metabolite, and one for amphetamines. Fifteen patients were found to have repeatedly negative urine drug screens for prescribed opioids despite being counseled on at least one occasion about the proper scheduling of their medication. The absence of the prescribed opiate was confirmed with GC/MS. Nine patients had repeatedly positive UTS for non-prescribed opioids, despite being counseled on at least one occasion that this was a violation of the medication agreement. Ten patients habitually obtained opioids from multiple providers (We did not consider the occasional use of the emergency department as a violation, but counseled patients against this practice unless clinically necessary). Two patients were found to have forged prescriptions, and one patient was diverting medications. All patients who violated the clinic opioid misuse policy were offered referral for counseling, but only two followed through, to our knowledge. Although not considered opioid misuse, eighteen percent had a UTS positive for cannabinoids at least once during the study period.

### Predictors of opioid misuse

Predictors of opioid misuse were examined in bivariate and multivariate analyses. In bivariate analyses (Table 3), misusers were more likely than non-misusers to have past cocaine abuse (68% vs 21%; p < 0.001), have a previous drug or DUI conviction (40% vs 11%; p < 0.001), be younger (48 years vs 54 years, p < 0.001), have past alcohol abuse (44% vs 23%; p = 0.004), or be male (59.7% vs. 38%; p = 0.005). Similar to cocaine abuse, the presence of cannabinoids on UTS obtained at any time during the 12 month follow-up period (33% vs 12%; p = 0.001) was a predictor of misuse. A previous drug or DUI conviction or multiple drug convictions were more strongly associated with misuse, with relative risks of 3.6, and 15.1, respectively. Race, income, education, depression score (CES-D), disability (PDI), and literacy score (REALM) were not associated with opioid misuse. There was no consistent correlation between pain scores and the risk of misuse, although misusers reported a higher intensity of *current *pain at baseline (Table 4).

In multivariate analyses (Table 5), age, self-reported histories of cocaine or alcohol abuse, drug or DUI convictions were shown to be the most powerful predictors of misuse (AUC, 0.827). The effect of a history of cocaine abuse was moderately strong (OR, 4.3; CI, 1.76 – 10.4). The odds ratios (OR) for drug or DUI convictions and a history of alcohol abuse were both 2.6. Age, though statistically significant in the model, did not clinically discriminate well between misusers and non-misusers. In the adjusted analyses, the average age was 53 years for misusers and 49 years for non-misusers. We performed analyses of the subset of opioid misusers who were not abusing stimulants (N = 37). The bivariate sub-analysis demonstrated general persistence of the statistical relationships seen in the entire sample (Table 6).

## Discussion

We identified predictors of opioid misuse in a cohort of opioid-treated patients with chronic pain who were enrolled in a primary care-based disease management program. Our program and study was not designed to make systematic substance abuse, dependence, or addiction diagnoses but rather to apply a working diagnosis of misuse that defined conditions under which opioids would be prescribed. The strongest predictors of misuse in the study population were self-reported histories of previous alcohol or cocaine abuse, or previous criminal drug or alcohol-related convictions. Age was also predictive, but the effect was not large. Gender, race, literacy, disability, and measures of socioeconomic status were not associated with misuse. The most frequent type of misuse involved the concurrent use of stimulants, usually cocaine. In a separate bivariate sub-analysis of patients with opiate misuse other than cocaine or amphetamines on UTS, the relationships between predictors and outcomes were similar, as the magnitudes of the odds ratios shown in Table 6 suggest. Our findings stand in contradistinction to other research that has found no predictive relationship between past alcohol and substance abuse and future opioid abuse in patients with chronic pain [45]. The pattern of drug misuse in the study population suggested the potential for multiple co-morbid diagnoses of substance abuse or dependence, placing these individuals at especially high risk of morbidity and mortality [46].

The limited clinical trials in the literature examining the use of opioids in the treatment of chronic pain do not identify factors that put chronic pain patients at risk for opioid misuse. They do not provide concrete guidance about how to select appropriate candidates for opioid therapy in a primary care setting. Although the incidence of misuse that we report is higher than that reported in other studies, many studies have not clearly defined their monitoring procedures to detect opioid misuse, have excluded patients *a priori *with significant mental illness (even major depression) or history of drug misuse [16], and have been conducted in specialty settings [18,47-49].

Some authorities have asserted that substance abuse and dependence are uncommon or rare consequences of opioid use for pain; however, the heterogeneity of the available evidence does permit accurate estimates of the prevalence or incidence of abuse in opioid-treated patients. One widely cited reference estimates opioid *addiction *at approximately 4 in 10,000 treated patients [50]. Such a low prevalence of misuse in opioid-treated patients, moreover, is inconsistent with epidemiological data that conservatively estimate the 12-month prevalence of drug misuse at 80 in 10,000 [51]. Pain specialty clinics have reported prevalences of dependence ranging from 3% to 17% [52]. In primary care, a retrospective study of two clinics documented *misuse *of opioid medications at 24% and 31%, respectively [53]. A study from Sweden suggests that abuse is common is patients with chronic pain. In that study, 414 hospitalized patients with chronic pain were systematically evaluated for substance abuse using the Substance Use Disorder Diagnostic Schedule based on the *Diagnostic and Statistical Manual of Mental Disorders, Third Edition*. Twenty-three percent of patients were found to have active drug abuse disorders [54]. In general, it is difficult to apply DSM-IV criteria for substance abuse or dependence in the context of prescription opioid use.

We chose the term *misuse *in our study because misuse encompasses behaviors with both medical and non-medical dimensions, whereas *abuse *more properly denotes the medical substance abuse or dependence disorders. The standard psychiatric definitions of abuse and dependence focus on tolerance and withdrawal which cannot be used to identify aberrant behavior in patients who are prescribed and regularly taking the medication that they may or may not be abusing as well. We adhered to published guidelines and literature that discourage opioid prescribing to patients with a history of previous or ongoing substance abuse. Stimulant-positive urines were considered evidence of, or proxy for, ongoing substance abuse and hence a contraindication to prescribing opioids. Evidence of stimulant abuse thus constituted opioid misuse as defined by our medication agreement but not opioid abuse or dependence per se. Also, we suspected that another subset of patients was procuring and diverting opioids for monetary gain as evidenced by the frequent finding of negative UTS in patients who reported they were using their medication as directed. These misusers might not receive substance abuse diagnoses. Based on consistently negative UTS, diversion of O/CS medications appears to be a common form of misuse encountered in our study. While the reasons for different forms of misuse were not qualitatively examined, the high street value of prescription opioids may have led to a temptation to sell them [55]. Alternatively, patients with negative UTS may have "used up" their prescriptions by taking their medication at a greater than agreed upon rate, although all patients found to have negative UTS asserted that they were taking their medications correctly, and UTS confirmation should have revealed their presence. In addition, we did not often witness the physiologic opioid withdrawal one would have expected in these patients.

We chose not to define a single positive cannabinoid test on UTS while receiving O/CS pharmacotherapy as an act of misuse that would result in sanction. We did, however, advise patients against marijuana use. Research in twins suggests that marijuana use is a risk factor for developing more severe and pervasive drug misuse disorders [56]. Our data suggest that marijuana users may be at higher risk of misuse and might require more vigilant monitoring.

Currently, most primary care settings have not organized care in a way that allows systematic evaluation of patients with chronic pain for either response to pharmacotherapy or misuse [23,30,57]. We believe that our pragmatic approach to monitoring opioid misuse based on the specific elements of the medication agreement can be replicated in primary care settings that do not have the resources to systematically evaluate patients for substance abuse or dependence. It provides a rational template for treating pain effectively and compassionately with opioids [31], while also offering providers reassurance that their actions are not contributing to the growing public health problem of prescription drug diversion and misuse.

Striking a balance between appropriate use of opioids and prevention of misuse is important for successful management of chronic pain. This study and others have found that the multidisciplinary disease management for chronic pain, can produce significant reductions in pain, improvements in depression and health-related quality of life through the establishment of a pain diagnosis and management plan [58,59]. Recent restrictions in the Drug Enforcement Administration regulations with regard to the provision of Schedule II controlled substances [60,61], along with rare but high-profile prosecutions of pain-treating physicians [62,63], have highlighted the need for continued care in prescribing these agents. Systematic approaches to pain management and detecting opioid misuse can reassure physicians that they can alleviate suffering with opioids without inviting criminal sanction or negatively impacting public health.

This study has several limitations. As noted above, the study population was drawn from referrals within a single, academic general internal medicine practice. As such, the sample may not be representative of all opioid-treated patients in primary care settings. Because we sought referrals of patients that were difficult to manage, the incidence of opioid or other drug misuse in this investigation may be higher than in other primary care or community-based populations of opioid-treated patients. Public information on drug offenses and DUI, while easily obtained online in North Carolina, is less accessible in other states. The initial assessment of prior or current drug misuse was based on self-report and clinical assessment rather than a structured diagnostic interview; better measurement may have allowed more accurate classification and assessment of risk. In addition, we did not inquire about histories of substance abuse other than alcohol and cocaine. Finally, we have limited data about the patients' outcomes after completing the study. Patients who were identified as committing misuse usually dropped out of the program, and we were unable to assess outcomes of pain, functional status, and mental health status once contact was lost.

## Conclusion

Identifying chronic pain patients at risk for opioid misuse remains a challenge. This study and other studies of chronic pain patients [52-54], suggest that the prevalence of any substance misuse may approach one-quarter of chronic pain patients receiving opioids. Opioid misuse was more common in patients with a self-reported history of alcohol or cocaine abuse. Previous criminal convictions for DUI or drug offenses predicted opioid misuse. Based upon these data, patients with a history of alcohol or cocaine abuse and alcohol or drug related convictions should be carefully evaluated and followed for signs of misuse if opioids are to be prescribed.

Additional prospective studies in primary care settings are needed to confirm these findings and to examine other potential predictors of opioid misuse. Also, better studies of interventions to reduce misuse of opioids and programs designed to effectively treat pain in patients with active substance abuse disorders are needed [64]. At the public health level, several states are considering legislation to allow better monitoring of prescriptions of controlled substances, such as state-wide registries, that may reduce some types of misuse, particularly the procurement of medication from multiple sources.

## Competing interests

The author(s) declare that they have no financial or non-financial competing interests.

## Authors' contributions

TJI developed the study design and intervention, administered surveys, directed pain management and drug misuse monitoring, assisted in the drafting, editing, and revision of the manuscript. PRC developed the study design and intervention, performed statistical analyses and drafted, edited, and revised the manuscript. CAH-S developed drug misuse monitoring protocols, provided expert consultation on clinical protocols using urine toxicological testing and edited the manuscript. RMM developed the study design, oversaw data management, and edited and revised the manuscript. JSP developed opioid misuse monitoring protocols, administered surveys, performed data management, edited the manuscript. NMP developed opioid misuse monitoring protocols, administered surveys, performed data management, edited the manuscript. BBS developed the study design, participated in data management, edited the manuscript. DAD provided statistical analytical support, and assisted in the drafting, editing, and revising the manuscript. MPP developed the study design, supervised overall conduction of the study, participated in data analysis, and assisted in the drafting, editing, and revision of the manuscript.

## Pre-publication history

The pre-publication history for this paper can be accessed here:



## Figures and Tables

**Table 1:** Patient Demographics

**Univariate Analysis (N = 196)**		
**Patient Demographics**	**Mean/%**	**SD (Range)**

Age	52	11 (26 – 85)
% Male	55	
% White	75	
% Completed High School	60	
% Disabled	57	
% Income < $20,000/year	85	
% Medicaid/Medicare	62	
% Uninsured	30	
% Smoker		
Current	57	
Ever	84	
% Ethanol Use:		
Current	19	
Ever	78	
% History of Ethanol Abuse	28	
% History of Cocaine Abuse	29	
% Receiving Opioids	96	
% Receiving Benzodiazepines	37	
% UTS Positive for cannabinoids over 1 year	18	
CES-D Depression Score	23.6	13 (0 – 54)
% Depression (CES-D > 15)	74	
% Severe Depression (CES-D > 22)	54	
PDI	45.2	13 (4 – 70)
REALM	51.2	19 (0 – 66)
% Drug Conviction in NC	12	
% DUI	11	
% Drug or DUI	20	
% Multiple Drug Convictions	4	
% Any Conviction	32	

**Table 2:** Serious Opioid Misuse (N = 196)

	**Number (%)**
**Opioid Misuse**	**62 (31.6 of total)**

Stimulants (Cocaine or Amphetamines)	25 (40.3)
Negative Urines	15 (24.2)
Doctor Collecting	10 (16.1)
Inconsistent Urines	9 (14.5)
Prescription Forgery	2 (3.2)
Diversion	1 (1.6)
Total	62 (100.0)

**Table 3:** Bivariate Analyses of All Opioid Misusers (N = 62)

	**Non-Misusers**	**Misusers**	**p-value**	**RR (95% CI)**
Age	54	48	< 0.001	
% Male	38	59.7	0.005	1.56 (1.16 – 2.11)
CES-D	22.5	26.0	0.080	
PDI	45.1	45.6	0.838	
REALM	50.7	53.5	0.492	
% Current Smoker	50	71	0.007	1.41 (1.12 – 1.79)
% History of Ethanol Abuse	23	44	0.004	1.95 (1.25 – 3.03)
% History of Cocaine Abuse	21	68	< 0.001	3.30 (2.17 – 4.99)
% Positive Urine Cannabinoids	12	33	0.001	2.70 (1.5 – 4.85)
% Multiple Drug Convictions	1	11	0.001	15.13 (1.90 – 120)
% Drug or DUI Convictions	11	40	0.001	3.60 (2.05 – 6.34)

**Table 4:** Baseline Pain Scores and Opioid Misuse (N = 196)

	**Non-Misusers**	**Misusers**	**p-value**
Worst Pain in Past Month	9.2	9.2	0.920
Least Pain in Past Month	4.3	4.5	0.536
Average Pain in Past Month	6.5	6.6	0.569
Pain Right Now	6.3	7.2	0.021

**Table 5:** Multivariate Analysis: Predictors of Opioid Misuse ^#^

Model*^%^	**Odds Ratio (95% CI)**	**p-value**
Age	0.95 (0.90 – 0.99)	0.027
Drug or DUI Conviction	2.58 (1.01 – 6.59)	0.030
History of Cocaine Abuse	4.30 (1.76 – 10.4)	0.001
History of Ethanol Abuse	2.60 (1.12 – 6.26)	0.048

# = Models were reduced using the Likelihood Ratio Test

% = Positive urine cannabinoid and history of cocaine use were strongly correlated.

**Table 6:** Bivariate Analysis: Predictors of Opiate Misuse for Subset of Patients with Misuse other than Stimulants on UTS (N = 37)

	**Non-Misusers**	**Misusers**	**RR (95% CI)**
Age	53.9	46.1	
% Male	38.1	59.5	1.97 (1.10–3.52)
% Positive Urine Cannabinoids	16.3	46.7	2.87 (1.68–4.88)
% History of Cocaine Abuse	15.5	47.8	3.10 (1.60–5.96)
% History of Ethanol Abuse	15.8	34.1	2.15 (1.20–3.85)
% Current Smoker	50.4	67.6	1.34 (1.013–1.78)
% Multiple Drug Convictions	20.4	75.0	3.68 (1.94–6.99)
% Drug or DUI conviction	17.9	42.3	2.36 (1.34–4.16)
